# Lemierre Syndrome With Streptococcus constellatus Bacteremia

**DOI:** 10.7759/cureus.50580

**Published:** 2023-12-15

**Authors:** Miles Thomas, Christopher J Peterson, Lauren N Mazin, Jonas Rawlins

**Affiliations:** 1 Internal Medicine, Virginia Tech Carilion School of Medicine, Roanoke, USA

**Keywords:** bacteremia, streptococcus constellatus, pulmonary emboli, septic thrombophlebitis, lemierre syndrome

## Abstract

Lemierre syndrome is characterized by thrombophlebitis of the internal jugular vein (IJV) secondary to bacterial pharyngitis or tonsillitis. Though antibiotic use has made this a rarer syndrome, it can nevertheless manifest in patients presenting with pharyngitis. Herein, we describe a 20-year-old male patient with no relevant medical history presenting with signs concerning for pneumonia and was ultimately diagnosed with Lemierre syndrome with *Streptococcus constellatus* bacteremia. Complications included IJV thrombus with presumed septic emboli to the lungs. The patient was discharged on ampicillin/sulbactam with plans to transition to amoxicillin/clavulanate.

## Introduction

Lemierre syndrome is classically characterized by bacterial invasion of the pharyngeal mucosa, often preceded by a bacterial or viral pharyngeal infection [1,2], leading to the development of internal jugular vein (IJV) thrombophlebitis and disseminated septic emboli [1-3]. The most frequent causative organism is *Fusobacterium necroforum*, an anaerobic gram-negative rod, which has become synonymous with the disease. However, various other bacteria have been isolated in cases of Lemierre syndrome and should be considered when beginning empiric therapy. We describe such a case here.

## Case presentation

A 20-year-old male with no significant past medical history presented to the emergency department with a four-day history of cough, shortness of breath, non-bloody diarrhea, non-bloody emesis, decreased appetite, body aches, sweats, fevers up to 103º F and significant fatigue. He also reported a recent sore throat which had resolved prior to presentation. No signs of neck or oropharyngeal pathology were noted by the emergency medicine team. During this initial encounter, he was noted to have a leukocytosis (12.5 K/uL), mild anemia (12.7 g/dL), and an elevated D-dimer level (5.8 FEU/mL)(Table 1). Although no remarkable findings were seen on the chest X-ray, a CT scan of the chest showed multifocal consolidative changes throughout the middle and bilateral lobes, with no evidence of deep vein thrombosis (DVT). The patient had performed several COVID-19 tests prior to presentation, all of which were negative. He was started on doxycycline for presumed community-acquired pneumonia and was sent home with instructions to return if symptoms worsened.

**Figure 1:**
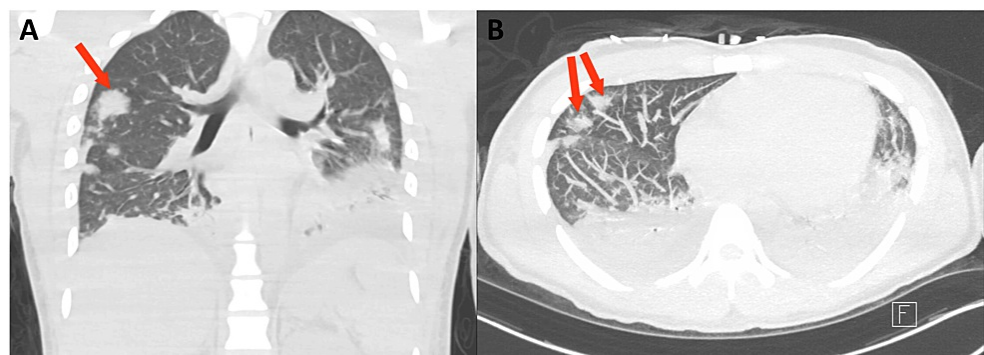
CT Chest with Pulmonary Septic Emboli CT chest without contrast showing pulmonary septic emboli (arrows) and small bilateral pleural effusions. Scattered airspace opacities are most prominent in the lower lobes. Findings are consistent with bilateral pneumonia.

Over the next 24 hours, the patient’s shortness of breath progressed and he returned to the emergency department, where labs revealed a worsening leukocytosis (16.2 K/uL) and an elevated pro-BNP (3292 pg/mL). A viral panel including testing for SARS-COV-2, influenza A/B, and RSV was negative. Hazy bilateral infiltrates were now evident on the chest X-ray. A CT scan of the chest continued to demonstrate bilateral multifocal infiltrates consistent with atypical pneumonia and concerning for possible septic emboli (Figure 1), ultimately concerning for sepsis. He was started on empiric antibiotic coverage with vancomycin, cefepime, and azithromycin. A transthoracic transesophageal echocardiogram observed a left ventricular ejection fraction of 50% without any vegetation. A CT of the head and brain with and without contrast was unremarkable and without signs of emboli. The following day, worsening bilateral nodular infiltrates were seen on a repeat chest X-ray. Laboratory results showed worsening leukocytosis (17.4 K/uL), anemia (hemoglobin 10.9 g/dL) with a normal haptoglobin and slightly elevated LDH (300 IU/L), thrombocytopenia (platelets 49 K/uL), elevated procalcitonin (27.70), and elevated ferritin (592.9 ng/mL). He was subsequently transferred to the intensive care unit for a higher level of care given the worsening pneumonia and risk of decompensation.

**Table 1:** Selected Lab during Illness Course ED = emergency department, ICU = intensive care unit, WBC = white blood cells, pro-BNP = pro-B-type natriuretic peptide

Test	Result				Reference Range
	Initial ED Visit	Subsequent ED Visit and Admission	Transfer and ICU Upgrade	Discharge	
WBC (K/uL)	12.5	16.2	17.4	7.6	4.0 – 10.5
Hemoglobin (g/dL)	12.7	12.5	11.2	12.0	13.0 – 16.0
Platelet Count (K/uL)	81	56	49	596	130 – 400
Lactic Acid (mmol/L)	1.6	1.8	0.9	-	0.5 – 2.0
Pro-BNP (pg/ml)	-	3292.0	1771.0	-	< 125

**Figure 2:**
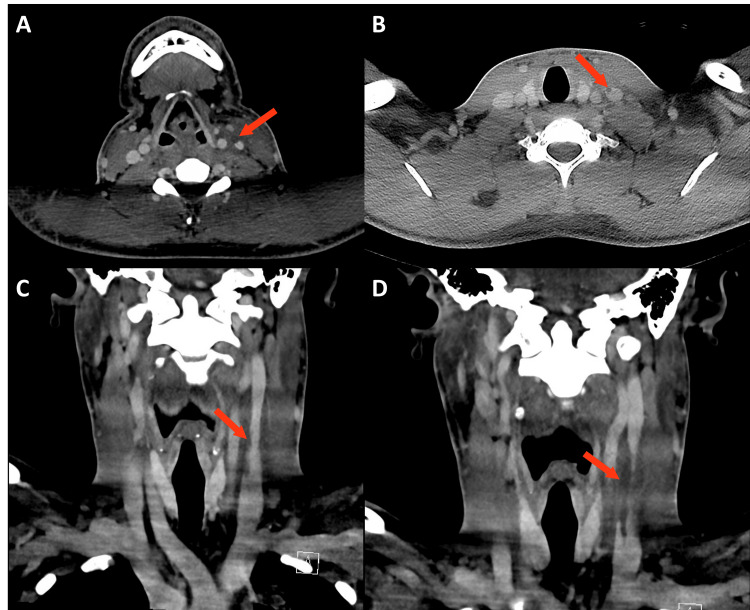
CT Soft Tissue Neck With Thrombus in the Left Internal Jugular Vein CT soft tissue neck with contrast showing a filling defect within the left internal jugular vein consistent with a moderate thrombus (arrows). Diffuse fat stranding is also noted throughout, suggesting a degree of anasarca.  No focally compressive phlegmons or abscesses are noted.

Upon presentation to the ICU, the patient was febrile (103.1 °F), tachycardic (122 bpm), tachypneic (RR 31), and with oxygen saturation at 94% on 2 liters nasal cannula. Physical exam was notable for bilateral cervical lymphadenopathy and bilateral wheezes throughout the upper lung fields. Oral examination was unremarkable, though sore throat prior to presentation was concerning for possible oral source of infection. Antibiotic coverage was subsequently changed to amoxicillin/clavulanic acid and doxycycline to cover atypical pneumonia, anaerobes, and tick-borne illnesses. The infectious diseases team was consulted and initiated an extensive work-up in addition to previous studies, given the infection of unknown etiology, elevated inflammatory markers, and thrombocytopenia (Table 2). Preliminary blood culture results identified *Streptococcus anginosus* group by Verigene. A CT scan of the neck with contrast demonstrated a thrombus in the left IJV (Figure 2). Antibiotics were then narrowed to ampicillin/sulbactam to cover Streptococcus species. Given the presence of septic emboli, anticoagulation was discussed with the infectious disease team and was ultimately decided against. A subsequent transesophageal echocardiogram noted an improved ejection fraction (60-65%) without any vegetations or intracardiac shunts. Finalized blood culture results showed revealed *Streptococcus constellatus*. At this time, findings were most consistent with a* S. constellatus* infection with multifocal pneumonia secondary to septic thrombophlebitis (Lemierre’s syndrome). 

**Table 2:** Infectious and Immunological Work-Up EIA = Enzyme Immunoassay, PCR = Polymerase chain reaction, Ab = Antibody, Ag = Antigen, CMV = Cytomegalovirus, EBV = Epstein Barr Virus, HIV = Human Immunodeficiency Virus, TB = Tuberculosis *Results are indeterminate for response to ESAT-6 and/or CFP-10 test antigens.

Test	Result	Reference Range
Blastomyces Ag (EIA)	None Detected	None Detected
CMV DNA (PCR)	Not Detected	Not Detected
Coccidioides Ag (EIA)	Not Detected	Not Detected
Cryptococcal Ag	Negative	Negative
EBV DNA (PCR)	Not Detected	Not Detected
Histoplasma Ag (EIA)	None Detected	None Detected
HIV-1/2 Ag/Ab (4^th^ Gen)	Non-Reactive	Non-Reactive
HIV-1 RNA (PCR)	Not Detected	Not Detected
Legionella Pneumophila Urine Ag	Negative	Negative
Monotest	Negative	Negative
Quantiferon TB Gold Plus	Indeterminate*	Negative
Nil	0.06 IU/mL	-
Mitogen-Nil	0.34 IU/mL	-
TB1-NIL	<0.00 IU/mL	-
TB2-NIL	<0.00 IU/mL	-
Tickborne panel		
*A. Phagocytophilum *DNA (PCR)	Not Detected	Not Detected
* Babesia microti* DNA (PCR)	Not Detected	Not Detected
* B. miyamotoi* DNA (PCR)	Not Detected	Not Detected
* Borrelia* spp DNA (PCR)	Not Detected	Not Detected
*Ehrlichia chaffeensis* DNA (PCR)	Not Detected	Not Detected
Viral Hepatitis		
Hepatitis A Ab (Total)	Reactive	Non-Reactive
Hepatitis A Ab (IgM)	Negative	Negative
Hepatitis B Surface Ag	Non-Reactive	Non-Reactive
Hepatitis B Surface Ab	<10.00 mIU/ml	<10.00 mIU/ml
Hepatitis C Ab	Non-Reactive	Non-Reactive
Immunoglobulin Panel		
IgM	74 mg/dL	50 – 300 mg/dL
IgG	760 mg/dL	600 – 1640 mg/dL
IgA	113 mg/dL	47 – 310 mg/dL

The patient subsequently began to defervesce, with decreasing frequency of fevers and improvement of his leukocytosis and other laboratory parameters, including resolution of thrombocytopenia. A repeat chest X-ray continued to show multifocal opacities consistent with septic emboli, but overall interval improvement in aeration. The patient was subsequently discharged with an additional three weeks of intravenous (IV) ampicillin/sulbactam, transitioning to oral amoxicillin/clavulanic acid for an additional three weeks.

## Discussion

Here we report a case of Lemierre syndrome in an otherwise healthy male without obvious signs of oropharyngeal involvement on initial presentation and initial findings consistent with pneumonia rather than septic thrombophlebitis. Lemierre syndrome was first reported by French physicians Courmont and Cade in 1900 [4] and was described by French bacteriologist Andre-Alfred Lemierre in 1936 [5,6]. The syndrome was far more prevalent in the “pre-antibiotic" era, where treatments were often limited to IJV excision or ligation [7]. Due to the widespread use of antibiotics, rates of Lemierre syndrome have declined so significantly that some have labeled it a “forgotten disease” [8]. However, in recent decades, the rates of Lemierre syndrome have increased [9,10], possibly due to reduced antibiotic use for pharyngitis and improvement in imaging techniques [11]. Incidence ranges from 3 to 14 cases per million persons, depending on the population studied [2,6]. Incidence rates are higher in adolescent and young adult patients [2]. Despite antibiotics, it remains a serious pathology with mortality rates as high as 18% [7].

Lemierre syndrome typically begins as an infection in the palatine tonsils and peritonsillar tissues [7], although other primary sources, such as sinuses, mastoid, oral, and auricular have been reported [12]. Invasion of the local tissue is not fully understood but may be due to an initial insult from viral or bacterial pharyngitis combined with bacterial-specific factors [6,7]. In many cases, there is no obvious inciting illness or injury, as observed in the case discussed here. Resulting bacteremia results in thrombophlebitis of the IJV [7]. From here, the thrombi embolize multiple tissues, most frequently the lungs, joints, or brain [13,14]. In rare cases, the thrombus may propagate to the subclavian or cranial sinuses [7]. Septic emboli in the lungs may result in abscesses, sterile effusions empyema, and cavitation [2,15]. Indeed, most investigations for Lemierre syndrome begin with chest X-rays, possibly due to associated lung pathology from septic emboli [7].

Lemierre syndrome occurs most frequently in healthy young adults, often males, in the second and third decades [2]. The reasons for this are unclear but may be due to the frequency of tonsillitis and pharyngitis in this demographic [16]. Diagnosis of Lemierre syndrome relies on diagnostic for identification of IJV thrombophlebitis, with CT the most common non-plain film modality [14]. Several authors note that a high degree of clinical suspicion is often needed to appropriately identify this condition [2], especially as the disease may initially be treated as pharyngitis or pneumonia. As mentioned by Lee et al., the presence of deep neck infections, septicemia, IJV thrombophlebitis, and signs of metastatic infection (such as septic emboli) should raise suspicion for Lemierre syndrome [2], especially if present in an otherwise healthy young adult. Early clinical signs and radiologic findings are crucial as the prolonged growth of anaerobic gram-negative bacteria, such as *F. necrophorum*, may delay diagnosis [2].

While *F. necrophorum *is the most frequent causative organism (81.7% of cases according to Chirinos et al. [13]), various bacteria have been isolated, though at far lower rates. The *S. anginosus *group (SAG) typically colonizes the reproductive and digestive tracts as well as the respiratory cavity and can cause visceral suppurative infections [17]. They are unique in their tendency to form abscesses and empyema. However, determining whether they are causal in a given infection can be difficult since they are resident oral cavity and respiratory tract flora [17]. A very small number of Lemierre syndrome cases involving the SAG species have been reported in the literature. As such, SAG appears to represent an uncommon group of pathogens in this syndrome [18]. Polymicrobial infections involve up to 30% of cases and, in many cases, are in combination with *F. necrophorum* [12].

Treatment involves empiric therapy, which is narrowed once bacteria are specified. Given the prevalence of *F. necrophorum* resistance of β-lactams, macrolides, fluoroquinolone, and aminoglycosides, β-lactamase-resistant antibiotics are often the recommended treatment [3]. Treatment length has not been established with randomized controlled trials but treatment for several weeks is often recommended [19]. Anticoagulation has been debated. Some have supported anticoagulation in cases where there are recurrent emboli, thrombus extension, or lack of improvement with antibiotic therapy [7], while others have opposed it due to the risk of bleeding. Multiple retrospective analyses have shown no benefit to anticoagulation. For example, a retrospective study of 394 patients found no difference in mortality [20]. Unfortunately, Lemierre syndrome can result in long-term complications; one study noted serious sequelae, such as neurologic deficits, in >10% of patients with Lemierre syndrome, possibly due to complications from septic emboli [6]. 

## Conclusions

In sum, we present a unique case of Lemierre syndrome with blood culture positive for *S. constellatus*. Clinicians should be cognizant of Lemierre syndrome as a cause of septic emboli in young, healthy adults and recognize that a variety of pathogens may be causative. In some cases, such as this one, patients may lack obvious clinical signs of oropharyngeal infection on initial presentation. As demonstrated here, infection and emboli of unknown origin may warrant imaging of the neck vasculature for thrombi.
